# Amorphous Ni*_x_*Co*_y_*P-supported TiO_2_ nanotube arrays as an efficient hydrogen evolution reaction electrocatalyst in acidic solution

**DOI:** 10.3762/bjnano.10.6

**Published:** 2019-01-07

**Authors:** Yong Li, Peng Yang, Bin Wang, Zhongqing Liu

**Affiliations:** 1 School of Chemical Engineering, Sichuan University, Chengdu 610065, Sichuan, P. R. China; 2 Engineered Multifunctional Composites (EMC), Knoxville, Tennessee 37934, USA

**Keywords:** electrocatalysis, electrodeposition, HER, NiCoP bimetallic phosphides

## Abstract

Bimetallic phosphides have been attracting increasing attention due to their synergistic effect for improving the hydrogen evolution reaction as compared to monometallic phosphides. In this work, NiCoP modified hybrid electrodes were fabricated by a one-step electrodeposition process with TiO_2_ nanotube arrays (TNAs) as a carrier. X-ray diffraction, transmission electron microscopy, UV–vis diffuse reflection spectroscopy, X-ray photoelectron spectroscopy and scanning transmission electron microscopy/energy-dispersive X-ray spectroscopy were used to characterize the physiochemical properties of the samples. The electrochemical performance was investigated by cyclic voltammetry, linear sweep voltammetry, and electrochemical impedance spectroscopy. We show that after incorporating Co into Ni–P, the resulting Ni*_x_*Co*_y_*P/TNAs present enhanced electrocatalytic activity due to the improved electron transfer and increased electrochemically active surface area (ECSA). In 0.5 mol L^−1^ H_2_SO_4_ electrolyte, the Ni*_x_*Co*_y_*P/TNAs (*x* = 3.84, *y* = 0.78) demonstrated an ECSA value of 52.1 mF cm^−2^, which is 3.8 times that of Ni–P/TNAs (13.7 mF cm^−2^). In a two-electrode system with a Pt sheet as the anode, the Ni*_x_*Co*_y_*P/TNAs presented a bath voltage of 1.92 V at 100 mA cm^−2^, which is an improvment of 79% over that of 1.07 V at 10 mA cm^−2^.

## Introduction

Significant research efforts have been invested in the electrochemical splitting of water using renewable energy to attempt to overcome the growing energy demands and associated environmental crisis [1–3]. In water splitting, the hydrogen evolution reaction (HER) is a fundamentally important process. This process involves the reduction of protons to form dihydrogen (2H^+^ + 2e → H_2_) with a thermodynamic potential of 0 V vs SHE. A major bottleneck for HER is the high overpotential associated with the process that takes place at a significant rate due to the high activation barrier and the sluggish multiple-proton-coupled electron transfer [4–6]. Noble metal Pt-based catalysts are widely used for HER to circumvent the overpotential hurdle, but their exorbitant cost and scarcity seriously limit their large-scale application. Hence, it is quite appealing to develop inexpensive and earth-abundance electrocatalysts with higher electrolytic efficiency and lower dynamic overpotential [7–8].

More recently, transition-metal phosphides (TMPs) have attracted great interest as efficient HER electrocatalysts, including Ni*_x_*P, MoP, CoP, FeP and Cu_3_P. These materials are significantly promising because of their abundance, remarkable stability and activity derived from their hydrogenase-like catalytic mechanism [9–14]. By adding an additional metal element to these mono-metal posphides, the electronic structure and surface properties of the phosphides can be intrinsically altered that may greatly improve the catalytic performance. Compared to mono-metal phosphides, some binary metal phosphides (MgFeP, FeNiP, NiCoP, etc.) demonstrate a superior electrochemical performance. Because the ternary phases provide a synergistic effect, these bi-metal phosphides provide good electrical conductivity and electronic structure [15–17]. Among the bi-metal phosphides, Ni–Co–P catalysts have been intensively investigated. The similar radii of Co and Ni have been shown to be favorable to form ternary TMPs rather than secondary-metal doped phosphides [18–20]. As exemplified by Fu et al., hierarchical whisker-on-sheet nanostructures of NiCoP/nickel foam presented a superior performance, giving overpotentials of 59 mV and 220 mV to obtain current densities of 10 mA cm^−2^ and 100 mA cm^−2^ in alkaline electrolyte for HER, respectively [21]. However, the preparation procedure is more complicated and not environmentally friendly and includes a hydrothermal reaction, phosphorization step and KOH activation. This brings some difficulties to large-scale industrial application.

Amorphous catalysts intrinsically contain more defect sites which probably work as active centers compared to the crystalline counterparts. A representative work is that by Zhang et al. where they synthesized Ni–Co–P/nickel foam electrodes via a facile electroless deposition [22]. The as-prepared electrode requires only a small overpotential of 107 mV and 125 mV to achieve current densities of 10 and 20 mA cm^−2^, respectively. Unfortunately, although the TMPs present excellent electrocatalytic activity in alkaline electrolytes [21–23], they are very unstable under acidic conditions. One effective way to improve their stability is with an appropriate support material. Compared to the nickel foam or other substrates [19,23–24], TiO_2_ nanotube arrays prepared by anodization are favorable for the loading of catalysts and the fast transfer of electrons from the electrode to the active sites owing to the large surface area and distinctive 3D well-ordered nanotube structure. Furthermore, the curved interface and confined space facilitate the formation of amorphous phases with more active catalytic sites and contribute to the stability of active components [25–26]. Accordingly, in this study, the TNAs work as the support material in the preparation of Ni*_x_*Co*_y_*P/TNA hybrid electrodes by a one-step electrodeposition process. The physiochemical and electrochemical properties of as-prepared Ni*_x_*Co*_y_*P/TNAs electrodes were investigated in detail. In acidic aqueous solution, the Ni*_x_*Co*_y_*P/TNAs electrodes presented enhanced electrocatalytic activity and robust stability after incorporating Co into NiP.

## Experimental

### Preparation of Ni*_x_*Co*_y_*P/TNA electrodes

The TiO_2_ nanotube arrays used here were prepared using an electrochemical anodization technique according to our previous work [25–26]. In a three-electrode system, the TNAs act as the working electrode, Pt as the counter electrode, Ag/AgCl (saturated KCl) as the reference, and a constant voltage (−1.2 V vs Ag/AgCl) was applied to the system and the duration of the electrodeposition was 200 s. The electrolyte (0.05 mol L^−1^ Ni(NO_3_)_2_ + 0.05 mol L^−1^ Co(NO_3_)_2_ + 0.1 mol L^−1^ NaH_2_PO_2_) pH was adjusted with 5% HCl to about 1.0. After electrodeposition, the working electrode was rinsed with deionized water, absolute ethanol, and then deionized water, and dried under blowing air. The sample was named Ni*_x_*Co*_y_*P/TNAs. A control sample Ni–P/TNAs was prepared in a similar fashion without adding Co(NO_3_)_2_ in the electrolyte.

### Sample characterization

The following analytic methods were applied to provide structural information on the Ni*_x_*Co*_y_*P/TNA samples: X-ray diffraction (XRD, X’Pert pro MPD, Philips) for crystallographic texture, scanning electron microscopy (SEM, JSM-5900 LV, JEOV) for micro-morphology, transmission electron microscopy (TEM, Tecnai G2 F20 S-TWIN) for microstructure, UV–vis diffuse reflectance spectroscopy (UV2100) for photoabsorption properties, X-ray photoelectron spectroscopy (XPS, Escalab 250Xi, Thermo Fisher, Al Ka X-ray source generated at 12 kV and 15 mA) for chemical composition, and energy dispersive spectroscopy (EDS, JSM-7500F) for single nanotube chemical composition.

### Electrochemical measurements

The electrochemical characteristics of the samples were evaluated using a CHI650E electrochemical workstation (Chenhua, Shanghai) including linear sweep voltammetry (LSV), cyclic voltammetry (CV), electrochemical impedance spectroscopy (EIS), and Tafel analysis at 25 °C. The three electrode system was constituted of the sample working electrode, a platinum counter electrode, a Ag/AgCl (saturated KCl) reference electrode, and 0.5 mol L^−1^ H_2_SO_4_ as the electrolyte. During the LSV, CV, and Tafel experiments, the scan rate was 5 mV s^−1^. During the EIS experiment, the frequency range was 10^−2^–10^5^ Hz and the applied bias was the open-circuit potential of the samples. The measured current was normalized by the geometrical area of the cathodes immersed in electrolyte solution. The obtained potential (vs Ag/AgCl) was converted RHE after imposing *iR*_s_ correction, using the following Equation 1:

[1] $$E_{\text{RHE}}=E_{\text{Ag/AgCl}}+0.059\times \text{pH}+E_{\text{Ag/AgCl}}^{0}\left(0.197\right)$$

## Results and Discussion

### Characterization of electrocatalysts

**Figure 1:**
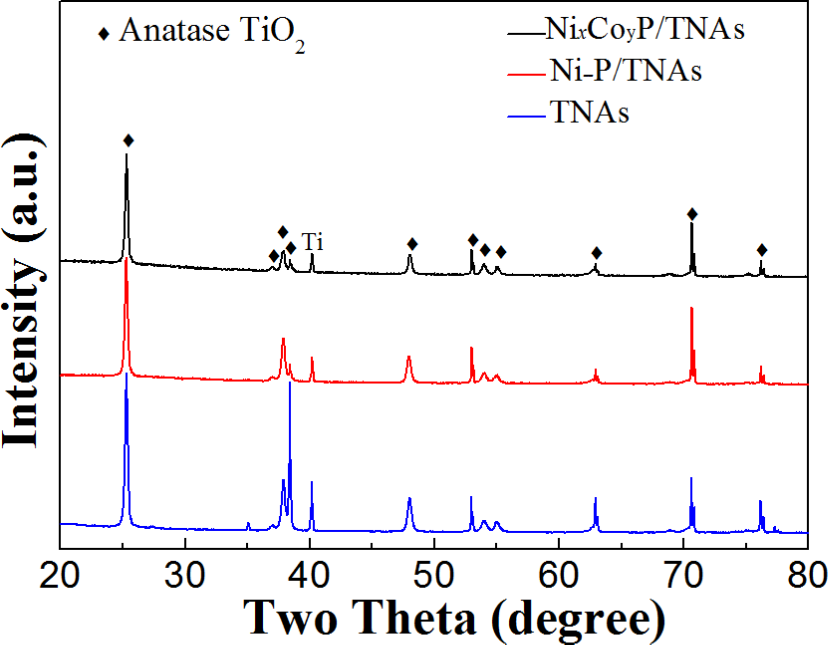
XRD patterns of the samples.

**Figure 2:**
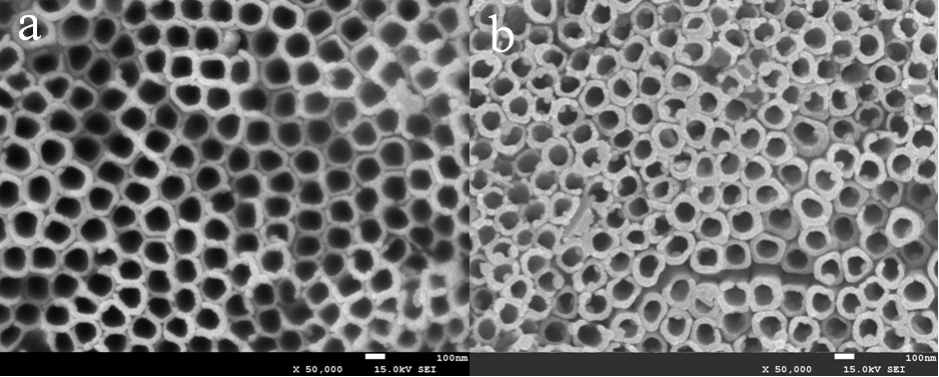
Top-view FE-SEM images of the samples. (a) TNAs, (b) Ni*_x_*Co*_y_*P/TNAs.

**Figure 3:**
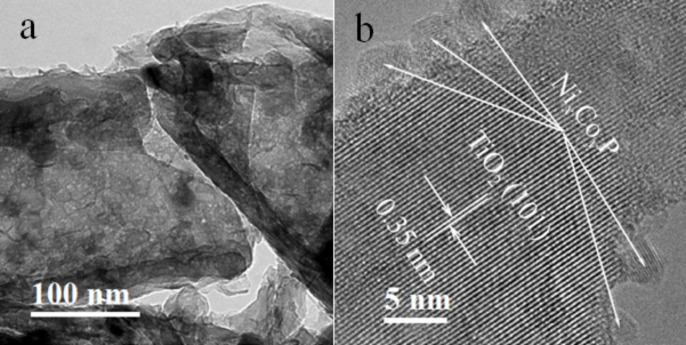
(a) TEM and (b) HR-TEM images of the Ni*_x_*Co*_y_*P/TNAs.

**Figure 4:**
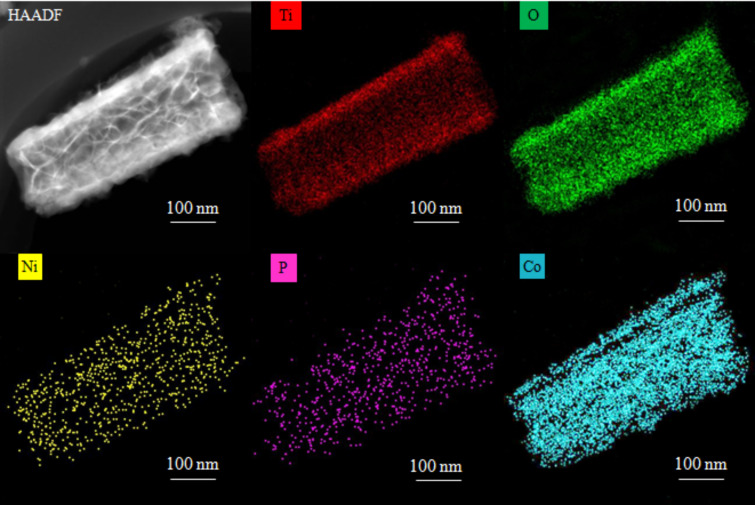
HAADF STEM image and EDS elemental maps of the Ni*_x_*Co*_y_*P/TNAs.

Figure 1 shows the wide-angle XRD patterns of the samples. All three samples displayed characteristic anatase TiO_2_ diffraction peaks of (101), (004), (200), (105), (211), (204), (116), and (215) (JCPDS card No. 21-1272) and the Ti peak at (101) [27]. No diffraction peaks related to Ni–P or NiCoP crystallites was found, illustrating that the crystallographic texture of the electrode samples was not altered by the electrodeposition of Ni–P or NiCoP. The intensity of the diffraction peaks follow the order: TNAs > Ni–P/TNAs > Ni*_x_*Co*_y_*P/TNAs. It is suggested that after electrodeposition, there was an amorphous deposit covering the TiO_2_ surface to dampen the anatase peak intensities. The top-view FE-SEM images of TNAs and Ni*_x_*Co*_y_*P/TNAs are shown in Figure 2. It is obvious that the openings of the TNAs were smooth with even wall thickness. After electrodeposition of NiCoP, the openings of sample Ni*_x_*Co*_y_*P/TNAs were coarse with apparent deposit attached. Figure 3 demonstrates the TEM and HR-TEM images of Ni*_x_*Co*_y_*P/TNAs. The lattice spacing of 0.35 nm is ascribed to anatase TiO_2_ (101) plane [28], and no lattice fringe that corresponds to NiCoP can be finely resolved. Combining the XRD and SEM results, we conclude that amorphous NiCoP particles of ≈6 nm were attached to TiO_2_ (101) phase, as shown in the upper left and square areas. The STEM-HAADF and corresponding EDS maps of single tube Ni*_x_*Co*_y_*P/TNAs are revealed in Figure 4. From the figure, the diameter of the TiO_2_ nanotube was determined to be about 150 nm with a chemical composition of Ti, O, Ni, Co, and P evenly distributed on the whole tube. The elemental intensity distributions of Ti and O were similar to one another, however the combinations Ni and P, Co and P, and Ni and Co did not have similar distributions. The distribution intensity of Ni is obviously higher than that of Co. It is possible that in addition to NiCoP, there might be other phases of Ni, Co, or P.

**Figure 5:**
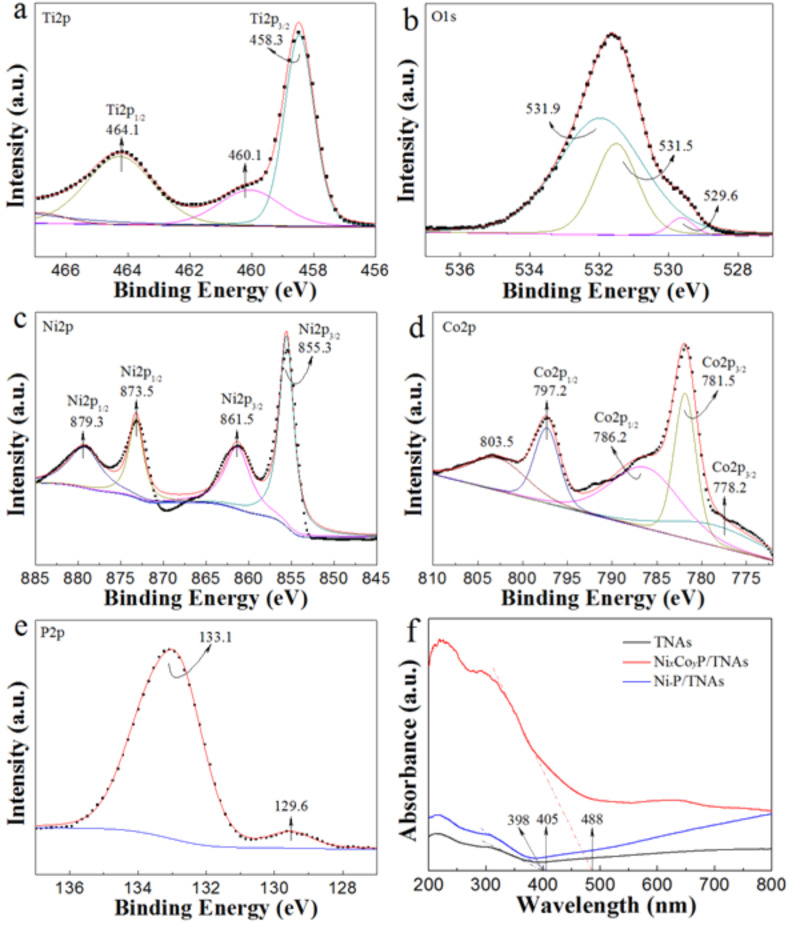
High-resolution XPS spectra of (a) Ti 2p, (b) O 1s, (c) Ni 2p, (d) Co 2p and (e) P 2p of the Ni*_x_*Co*_y_*P/TNAs. (f) UV–vis diffuse reflection absorbance spectra of the samples.

To further probe the surface chemical composition and valence states in the Ni*_x_*Co*_y_*P/TNAs, we conducted X-ray photoelectron spectroscopy measurements. In Figure 5a, TiO_2_ shows two peaks, the Ti 2p_3/2_ peak at 458.3 eV and Ti 2p_1/2_ at 464.1 eV, along with a satellite peak at 460.1 eV. The O 1s peaks at 531.5 and 529.6.5 eV are assigned to O in O_2_ and TiO_2_, respectively. The peak at 855.3 eV for Ni 2p_3/2_ can be ascribed to Ni^δ+^ in Ni−P bonds, positively shifted relative to that of metallic Ni (852.3 eV) (Figure 5c). The Ni 2p_3/2_ peak at 861.5 and Ni 2p_1/2_ peak at 879.3 eV are assigned to the Ni 2p satellite peaks [15,29–31]. In Figure 5d, the Co 2p_3/2_ peak at 778.2 is assigned to metallic Co, and the Co 2p_3/2_ peak at 781.5 and Co 2p_1/2_ peak at 797.2 eV can be ascribed to Co^δ+^ and Co^3+^ ions in NiCoP, respectively. The broad peaks at 786.2 (2p_3/2_) and 803.5 eV (2p_1/2_) are assigned to the Co 2p satellite peaks [21,32]. In the high-resolution P 2p spectrum of Figure 5e, the binding energy at 129.6 eV is close to the binding energy of P 2p_3/2_, assigned to metal–P bonds in NiCoP. The peak at 133.1eV can be ascribed to the oxidized phosphorus species by contact with air [21,33–35]. The binding energy of 129.6 eV is slightly lower than that of elemental P (130.0 eV), which suggests the P is partially negatively charged (P^δ−^) [36]. Given the probing depth of 3 nm for XPS measurements, the NiCoP amorphous phase in Ni*_x_*Co*_y_*P/TNAs presents a molar mole ratio of 10.82: 2.21:2.82, giving *x* = 3.84 and *y* = 0.78. According to the XPS results, polyvalent interactions of Ni, Co and P heteroatoms are suggested. In this complex material, both Ni and Co carry a partially positive charge (δ^+^) whereas P carries a partially negative charge (δ^−^), suggesting a small electron density transfer from Ni and Co to P [37]. This charged structure is very beneficial for improving surface activity toward HER.

A critical means to improve the charge transfer of HER is to enhance the conductivity of the electrocatalysts. Doping or hybridization to form a heterojunction can lower the band gap of the material thus augment the conductivity. The material band gap can be calculated by measuring the optical absorption edge in UV–vis DRS, shown in Figure 5f. It is observed that the absorption edge showed a red shift after electrodeposition of Ni–P and NiCoP. The absorption edges are 398, 405, and 488 nm for TNAs, Ni–P/TNAS, and Ni*_x_*Co*_y_*P/TNAs, corresponding the band gaps of 3.12, 3.06, and 2.54 eV, respectively. Sample Ni*_x_*Co*_y_*P/TNAs had a band gap 0.52 eV lower than that of Ni–P/TNAs. This indicates that the binary-metal phosphides synthesized via electrodeposition provide a higher conductivity in the material.

### Electrochemical activity

**Figure 6:**
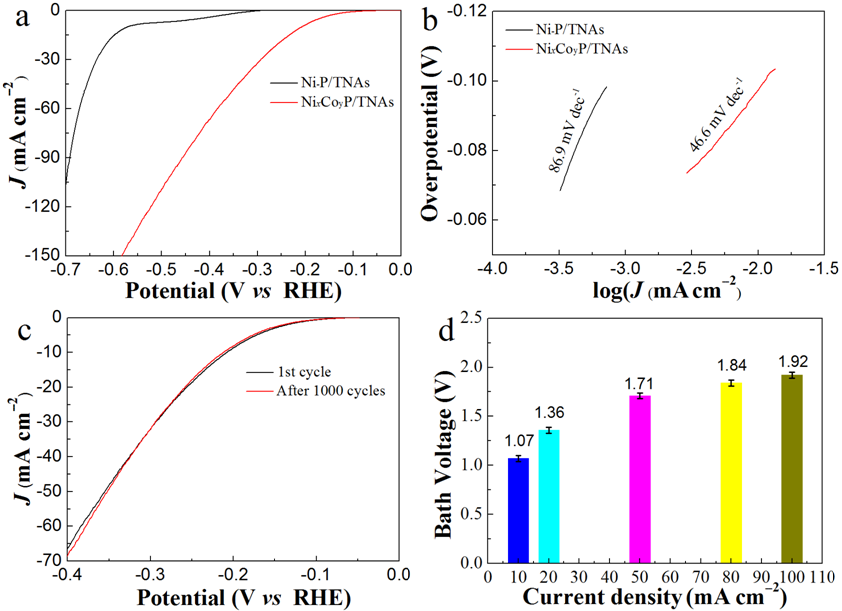
(a) Current–voltage characteristic plots and (b) Tafel plots of the samples. (c) Current–voltage characteristics during durability tests and (d) bath voltages at various current densities for the two-electrode system with Ni*_x_*Co*_y_*P/TNAs as a cathode.

The electrochemical properties of the samples are shown in Figure 6, including LSV, CV, Tafel curves, bath voltage histograms, and cycling stability characteristics. In Figure 6a, the activity of Ni*_x_*Co*_y_*P/TNAs is much higher than that of Ni–P/TNAs. The onset hydrogen evolution potential (defined as the potential at a current density of −0.1 mA cm^−2^) at −10 and −20 mA cm^−2^ of Ni*_x_*Co*_y_*P/TNAs were −65, −209, and −257 mV, respectively. These values are 235, 363, and 359 mV lower than that of Ni–P/TNAs of −300, −572, and −616 mV, respectively. It should be noted that the hydrogen doping may occur due to the small radius of the hydrogen atom when measuring the electrocatalytic activity of Ni*_x_*Co*_y_*P/TNAs. Generally speaking, hydrogen doping increases electrical conductivity and enhances electron transfer. Thus the electrocatalytic activity is improved to some extent. Figure 6b illustrates the Tafel curves of the Ni*_x_*Co*_y_*P/TNAs electrode. The Tafel slope of this electrode is 46.6 mV dec^−1^, which is 40.3 mV dec^−1^ lower than that of Ni–P/TNAs at 86.9 mV dec^−1^. For HER in acidic electrolyte, the theoretical Tafel slopes are 120, 40, and 30 mV dec^−1^, corresponding to the Volmer step, Heyrovsky step, and Tafel step, respectively. A Tafel slope of 46.6 mV dec^−1^ indicates that hydrogen evolution occurred via a fast discharge reaction (H_3_O^+^ + e^−^ + cat = cat-H + H_2_O) and thereafter a rate determining (ion + atom) reaction (H_3_O^+^ + e^−^ + cat-H = cat + H_2_ + H_2_O), that is, the Volmer–Heyrovsky mechanism [38–39]. A comparison was given with published data in Supporting Information File 1, where Table S1 and shows that NiCoP catalysts present lower overpotentials in alkaline electrolyte than those in acidic solution. The Ni*_x_*Co*_y_*P/TNAs electrode gives a lower activity than the electrode without the titanium dioxide carrier, which may be related to the low conductivity of titanium dioxide. Thus the electrocatalytic activity can be improved effectively by improving the conductivity of the TNA support.

In electrochemical HER, the bath voltage is an important parameter determining the energy consumption of the process. At a certain current density, the bath voltage is proportional to the electric energy consumption. In Figure 6d, the bath voltage was only 1.07 ± 0.03 V at hydrogen evolution current density of −10 mA cm^−2^ in the two-electrode system of Ni*_x_*Co*_y_*P/TNAs as the cathode and Pt sheet as the anode. A bath voltage of 1.71 V at a current density of 50 mA cm^−2^ is comparable to that of the NiCoP/foam nickel electrode [21]. It is noticeable that with increasing current density, the bath voltage does not rise in a linear pattern. A bath voltage of 1.92 V at 100 mA cm^−2^ is only 79% higher than that of 1.07 V at 10 mA cm^−2^. This demonstrates the excellent electrocatalytic activity of Ni*_x_*Co*_y_*P/TNA electrodes in acidic conditions. Other than the high electrocatalytic activity, the electrochemical stability is another critical parameter for electrodes in practical applications. The electrochemical activity of the Ni*_x_*Co*_y_*P/TNAs suffered a negligible decrease after 1000 cycles at a scan rate of 100 mV s^−1^ (Figure 6d). This shows a high stability of this electrode.

**Figure 7:**
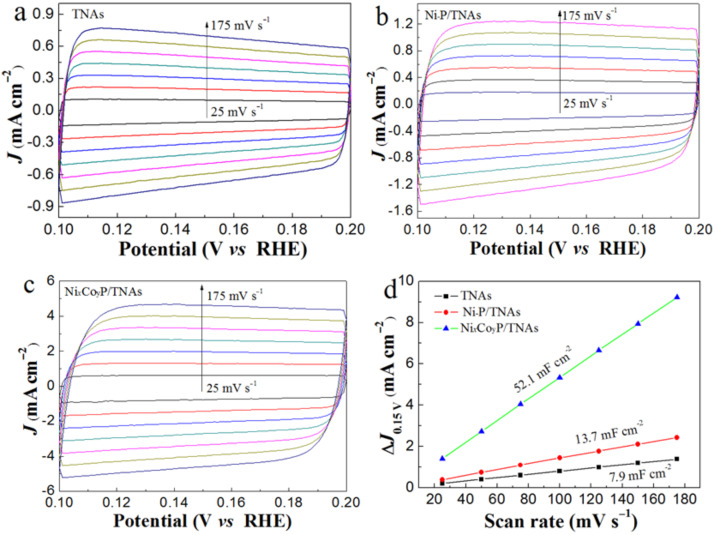
Cyclic voltammograms of (a) TNAs, (b) Ni–P/TNAs and (c) Ni*_x_*Co*_y_*P/TNAs at various scan rates (25–175 mV s^−1^), and (d) corresponding current density scan rate curves to estimate the *C*_dl_ and relative electrochemically active surface area.

For electrocatalytic reactions, the active site density is proportional to the reaction rate under certain conditions. The higher the density of exposed active sites, the faster the reaction rate. The active site density is related to the double-layer capacitance of the electrode surface without Faradic current and corresponds to the effective electrochemical surface area (ESA). Therefore the magnitude of the double-layer capacitance can be used to estimate the ESA. To estimate the effective ESA, we measured the electrochemical double-layer capacitances (*C*_dl_) using the CV method [22,40–41]. The scan rates during the CV measurements were set in the range of 25–175 mV s^−1^ (step by 25 mV s^−1^), electrode potential range of 0.1–0.2 V vs RHE. In Figure 7a–c, the CV curves are shown as zero-symmetric, rectangular curves against current density. This illustrates the double-layer capacitance nature of the electrode in this potential range and good reversibility. Figure 7d shows the double-layer capacitance of the Ni*_x_*Co*_y_*P/TNAs electrode to be 52.1 mF cm^−2^, which is 2.8 and 5.6 times that of Ni–P/TNAs (13.7 mF cm^−2^) and TNAs (7.9 mF cm^−2^). The incorporation of Co into the Ni–P formed amorphous binary-metal phosphides that are beneficial for the improvement of the electrocatalytic active site density, and thus the electrocatalytic activity.

**Figure 8:**
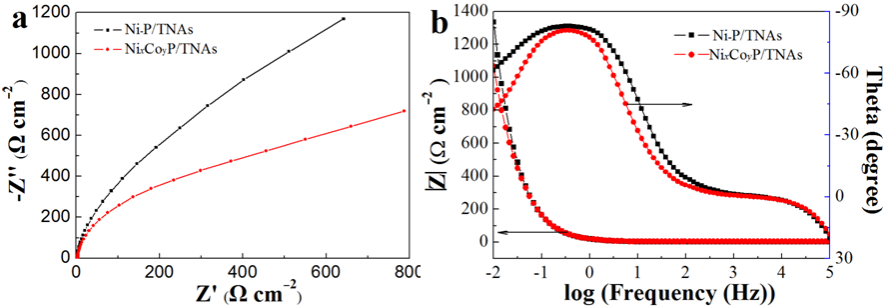
(a) Nyquist curves and (b) Bode plots of the samples.

The Nyquist and Bode plots are displayed in Figure 8. In the Nyquist plot, the arc radius of the high-frequency section corresponds to the impedance of charge transfer between electrolyte and the catalyst surface, and the ones of the low-frequency area correspond to the impedance of charge transport inside the electrode [15,42–44]. In Figure 8a, the Nyquist curves are shown as two arcs with different radius in the high and low frequency, suggesting that the catalytic reaction was limited by the charge transfer step. The arc radii of the high and low frequency sections of sample Ni*_x_*Co*_y_*P/TNAs are smaller than that of Ni–P/TNAs, suggesting that the NiCoP hybrid enhanced the charge transfer inside the electrode and between the electrolyte and catalyst surface. The Bode plots (Figure 8b) show that for the two samples, the total impedance (|*Z*|) is nearly equivalent at high frequency, while at low frequency, the impedance of Ni*_x_*Co*_y_*P/TNAs is lower than that of Ni–P/TNAs. This indicates that after incorporating Co into Ni–P, the main contribution is to improve the transmission of electrons inside the electrode, in agreement with a higher conductivity of Ni*_x_*Co*_y_*P/TNA confirmed by UV–vis diffuse reflection spectra. Both the CV and EIS results exemplify the high electrocatalytic activity of the Ni*_x_*Co*_y_*P/TNAs electrode, in accordance with the aforementioned electrochemical experiment results.

## Conclusion

The binary-metal phosphide hybrid electrode Ni*_x_*Co*_y_*P/TNAs was synthesized through the one-step electrodeposition of Ni, Co, and P under a constant voltage. Experimental results demonstrate that the NiCoP deposit was in amorphous phase with a diameter of ≈6 nm. The incorporation of Co into the binary Ni–P system formed the amorphous ternary NiCoP HER electrocatalyst. The catalyst showed a high electrochemically active center density that benefited the electron transfer within the electrode and between electrolyte and electrode surface. The electrocatalytic activity of the HER was thus improved. In the two-electrode system using Ni*_x_*Co*_y_*P/TNAs as the cathode, the bath voltage was only 1.07 V at hydrogen evolution current density of −10 mA cm^−2^, indicating superb electrocatalytic activity. The electrochemical stability of the electrode was proved via continuous cycling measurements.

## Supporting Information

File 1 Comparison of the overpotentials (vs RHE) between the references and this work.
