# Supplementary material for: Amorphous NixCoyP-supported TiO2 nanotube arrays as an efficient hydrogen evolution reaction electrocatalyst in acidic solution
Source: Beilstein J Nanotechnol. 2019 Jan 7;10:62–70. doi: 10.3762/bjnano.10.6 (PMC6334790; doi:10.3762/bjnano.10.6)
Supplement: File 1 — Comparison of the overpotentials (vs RHE) between the references and this work. [file Beilstein_J_Nanotechnol-10-62-s001.pdf]

BEILSTEIN JOURNAL OF NANOTECHNOLOGY

Supporting Information

for

Amorphous Ni_xCo_yP-supported TiO_2 nanotube arrays as an efficient hydrogen evolution reaction electrocatalyst in acidic solution

Yong Li, Peng Yang, Bin Wang and Zhongqing Liu

Beilstein J. Nanotechnol. **2019,** *10,* 62–70. doi:10.3762/bjnano.10.6

Comparison of the overpotentials (vs RHE) between the references and this work

License and Terms: This is a supporting information file under the terms of the Creative Commons Attribution License (http://creativecommons.org/licenses/by/4.0). Please note that the reuse, redistribution and reproduction in particular requires that the authors and source are credited.
The license is subject to the *Beilstein Journal of Nanotechnology* terms and conditions: (https://www.beilstein-journals.org/bjnano)

Table S1: Comparison of the overpotentials (vs RHE) between the references and this work.

**Catalyst**	**Substrate**	**Electrolyte**	**η_0 (mV)**	**η_{10} (mV)**	**η_{20} (mV)**	**η_{100} (mV)**	**Tafel slope ($mV\cdot dec^{-1}$)**	**Reference**
Ni-Co-P	NF	1 M KOH		107	125		62	[1]
NiCoP@Cu_3P	CF	1 M KOH		54			73	[2]
HWS NiCoP	NF	1 M KOH		59		220	90	[3]
NiCoP	CNT/NF	1 M KOH	69	80		173	62	[4]
$Ni_{2-x}Co_xP$	RGO	0.5 M H_2SO_4	31	42	55		45.2	[5]
NiCoP NPs		0.5 M H_2SO_4	42	97		158	52	[6]
Nest-like NiCoP	CC	0.5 M H_2SO_4		48		137	38.5	[7]
		1 M KOH		62		158	68.2	
H-NiCoP	NF	1 M KOH		44			38.6	[8]
NiCoP	NF	1 M KOH		32			37	[9]
**Ni_xCo_yP**	**TNAs**	**0.5 M H_2SO_4**	**65**	**209**	**257**		**46.6**	**this work**

References

[1] Yang,Q.P.; Lv, C.C.; Huang,Z.P.; Zhang.C. *Int. J. Hydgrogen Energy* **2018,** *43,* 7872–7880.

[2] Ma, X.;Chang, Y.; Zhang, Z.; Tang, J. *J Mater. Chem. A* **2018,***6,*2100–2106.

[3] Cai, Cai, Z.C.; Wu, A.P.; Yan,H.J.;Xiao, Y.L.; Chen,C.F.;Tian,C.G.; Wang,L.; Wang,R.H.; Fu, H.G. *Nanoscale* **2018,** *10,* 7619–7629

[4] Wu. J.;Ge, X.; Li, Z.; Cao, D.; Xiao, J. *Electrochemia Acta* **2017,** *252,* 101–108.

[5] Li, J.; Yan, M.;Zhou, X.; Huang Z.Q.; Xia, Z.; Chang, C.R.; Ma,Y.Y. *Adv. Funct. Mater.* **2016,** *26,* 6785–6796.

[6] Wang, C.; Jiang, J.;, Ding, T.; Chen, G.;Xu, W.;Yang, Q. *Adv. Mater. Interfaces* 2016,*3,*1500454.

[7] Du, C.;Yang, L.; Yang,F.; Cheng, G.; Luo, W. *ACS Catal*. **2017,***7,*4131–4137.

[8] Liu, C.; Zhang, G.; Yu, L,.; Qu, J.; Liu, H. *Small* **2018,** *14,*1800421.

[9] Li, Y.; Liu, J.; Chen, C.; Zhang, X.; Chen, J. *ACS Appl. Mater. Interf.* 2017, 9,5982–5991.
